# An asymmetric attraction model for the diversity and robustness of cell arrangement in nematodes

**DOI:** 10.1242/dev.154609

**Published:** 2017-12-01

**Authors:** Kazunori Yamamoto, Akatsuki Kimura

**Affiliations:** 1 Department of Genetics, SOKENDAI (The Graduate University for Advanced Studies), Mishima 411-8540, Japan; 2 Cell Architecture Laboratory, Structural Biology Center, National Institute of Genetics, Mishima 411-8540, Japan

**Keywords:** Embryogenesis, Diversity, Robustness, *C. elegans*, Computer modeling, Cadherin

## Abstract

During early embryogenesis in animals, cells are arranged into a species-specific pattern in a robust manner. Diverse cell arrangement patterns are observed, even among close relatives. In the present study, we evaluated the mechanisms by which the diversity and robustness of cell arrangements are achieved in developing embryos. We successfully reproduced various patterns of cell arrangements observed in various nematode species in *Caenorhabditis elegans* embryos by altering the eggshell shapes. The findings suggest that the observed diversity of cell arrangements can be explained by differences in the eggshell shape. Additionally, we found that the cell arrangement was robust against eggshell deformation. Computational modeling revealed that, in addition to repulsive forces, attractive forces are sufficient to achieve such robustness. The present model is also capable of simulating the effect of changing cell division orientation. Genetic perturbation experiments demonstrated that attractive forces derived from cell adhesion are necessary for the robustness. The proposed model accounts for both diversity and robustness of cell arrangements, and contributes to our understanding of how the diversity and robustness of cell arrangements are achieved in developing embryos.

## Abstract (precis)

**Summary:** The cell arrangement pattern at the four-cell stage in nematode embryos is both diverse and robust. A numerical model that incorporates attractive, as well as repulsive, forces was constructed to explain this diversity and robustness.

## INTRODUCTION

In multicellular organisms, nearby cells communicate with each other by sending and receiving signals mediated by their surface molecules (Alberts et al., 2008). The cell arrangement pattern, which refers to the pattern of cell–cell contacts, is important for development and homeostasis of an organism. During embryogenesis, specific cell arrangement patterns are established, and specific cell–cell contacts in the patterns define cell fate and body plan (Gilbert, 2016). Although the importance of the cell arrangement pattern is well studied, the mechanisms by which specific arrangements are established are not fully understood.

**Fig. 1.**
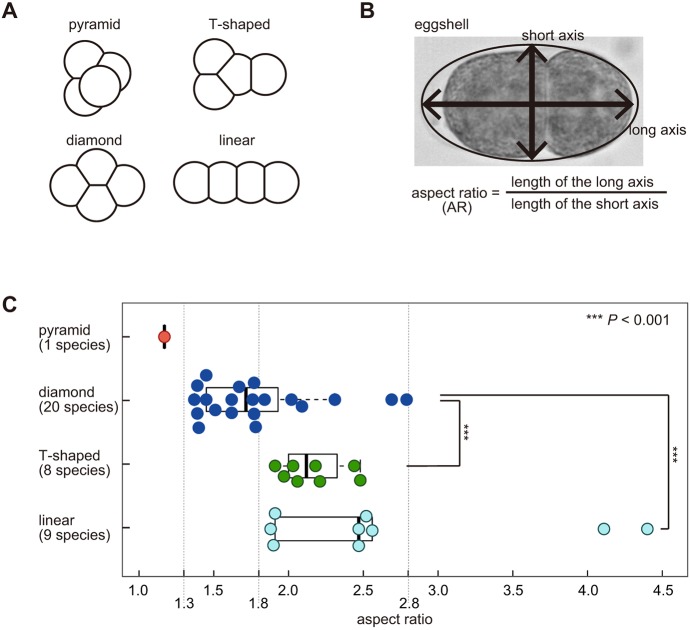
**Cell arrangement patterns in various nematode species.** (A) Classification of the cell arrangement patterns: Depending on the cell–cell contact, the patterns at the four-cell stage are classified into ‘pyramid’, ‘diamond’, ‘T-shaped’, or ‘linear’ types. (B) The AR was calculated as the length of the long axis divided by that of the short axis of the eggshell. (C) Bee swarm plot and box plot of the AR depending on the cell arrangement pattern (red, pyramid type; blue, diamond type; green, T-shaped type; cyan, linear type) in embryos of various nematode species; all data are summarized in Table S1. The box represents the 25-75th percentiles, and the median is indicated. The upper whisker shows the lower of the maximum or the upper quartile plus 1.5 times the inter-quartile range (IQR). The lower whisker shows the higher of the minimum or the lower quartile minus 1.5 times the IQR. Asterisks represent statistical significance as determined by Wilcoxon's rank-sum test. ***P<0.001 versus diamond arrangement.

In this study, we investigated the mechanical basis of the diversity and robustness of cell arrangement patterns by using four-cell stage nematode embryos as models. The cell arrangement patterns of nematode species at the four-cell stage show a high degree of diversity (Goldstein, 2001). The diversity of cell arrangement patterns is often explained by a variation in the orientation and position of the mitotic spindle (Akiyama et al., 2010; Pierre et al., 2016), particularly in four-cell-stage nematode embryos (Schulze and Schierenberg, 2011). However, the mitotic spindle is not the sole determinant of cell arrangement. Following cell division, cells move and change their arrangement depending on their interactions with other cells in a confined space. In the nematode embryo, this confined space is defined by the eggshell (Olson et al., 2012). At the four-cell stage, the *Caenorhabditis elegans* embryo generally acquires a ‘diamond’ type of cell arrangement (Fig. 1A) inside the eggshell, and a ‘T-shaped’ type (Fig. 1A) when the eggshell is removed (Edgar et al., 1994). Interestingly, eggshell shapes also show diversity among nematode species (Goldstein, 2001). We noticed that there was a correlation between eggshell shapes and cell arrangement patterns. We thus hypothesized that the diverse patterns of cell arrangements are produced by the diverse shapes of eggshells. The effect of eggshell shape on the pattern of cell arrangement had not been previously examined; therefore, in the present study, we attempted to alter the shapes of *C. elegans* eggshells to assess whether eggshell shape represents a source of diversity in cell arrangement patterns.

While cell patterns are diverse, individual species often acquire a specific pattern reproducibly (Gilbert, 2016; Schulze and Schierenberg, 2011). Such a robust pattern is critical for embryo development. Specific cell–cell contacts and their roles in development are well studied in *C. elegans*. At the four-cell stage, blastomeres acquire the diamond-type arrangement, in which two pairs of cells (EMS and P2, or ABp and P2 cells) contact each other (Fig. S1A) and send signals mediated by Wnt-Frizzled or Notch-Delta pathways, both of which are critical for establishment of the dorsal-ventral embryo axis (Gönczy and Rose, 2005). Changes in the arrangement pattern have deleterious effects on the embryo (Goldstein, 1992; Kemphues et al., 1988). To date, the robustness of cell arrangements has not been examined in a systematic manner. In this study, we examined how robust the diamond-type arrangement is to deformation of the eggshell in *C. elegans* embryos.

Mechanistic bases for the diversity and robustness of cell arrangements may be understood by constructing theoretical models. A good mechanical model that accounts for the diamond-type of cell arrangement has been reported previously (Fickentscher et al., 2013). The model assumes two types of repulsive forces: a repulsive force between cells, and a repulsive force between a cell and the eggshell. The model successfully reproduced both the position and trajectory of cells, up to the 12-cell stage for wild-type embryos (Fickentscher et al., 2013). Repulsive forces are commonly assumed to underlie the patterns of cell arrangements in various species (Akiyama et al., 2010; Kajita et al., 2003; Pierre et al., 2016; Zammataro et al., 2007). Such repulsive forces can be provided by the surface tension of the cell (Fujita and Onami, 2012). However, it has not been examined whether the previously reported model based on repulsive forces also accounts for the diversity and robustness of cell arrangements.

In this study, we focused on embryo deformation as a mechanical perturbation to investigate the diversity and robustness of cell arrangements. The purposes of this study were: (1) to test whether the shape of the eggshell accounts for the diversity of cell arrangement patterns in four-cell nematode embryos, (2) to characterize the robustness of the diamond pattern of *C. elegans* against deformation, (3) to construct a theoretical model to account for the diversity and robustness of cell arrangement, and (4) to elucidate the molecular basis of the model.

## RESULTS

### Eggshell shape and cell arrangement pattern are correlated in various nematode species

To examine whether the eggshell shape is related to the diversity of cell arrangements at the four-cell stage, we investigated the correlation between eggshell shapes and cell arrangement patterns in various nematode species. Based on images in published reports (Goldstein, 2001; Schulze and Schierenberg, 2011), the patterns of cell arrangements at the four-cell stage were classified into ‘diamond’, ‘pyramid’, ‘T-shaped’ or ‘linear’ types, which are defined by cell–cell contacts (Fig. 1A). We quantified the eggshell shape on the basis of the aspect ratio (AR), which is calculated by dividing the length of the long axis by that of the short axis of the eggshell (Fig. 1B), and associated them with the pattern of cell arrangement (Table S1). For *Diploscapter coronata* (Lahl et al., 2009) and *Aphelenchoides besseyi* (Yoshida et al., 2009), we imaged the embryos (Table S1). We then examined the relationship between the ARs and the cell arrangement patterns (Fig. 1C). The arrangements tended to change from the pyramid- or diamond- to T-shaped- or linear-type as the AR increased, supporting the notion that the diversity in eggshell shapes represents a source of the diversity in the cell arrangement pattern. The pyramid-type of cell arrangement was observed at low ARs (AR=1.2). The diamond-type was observed in 100% of the species for ARs from 1.3 to 1.8. For ARs over 4.0, only the linear type was observed.

Shape was found not to be the sole determinant of cell arrangement as different patterns were observed for similar AR values. For ARs from 1.8 to 2.8, the diamond, T-shaped and linear types were observed, depending on the species (Fig. 1C). This diversity, which is independent of the AR, may be caused by differences in other conditions such as the orientation of cell division (Schulze and Schierenberg, 2011). For example, in embryos with the linear-type arrangement at the four-cell stage, cells at the two-cell stage both divide along the long axis of the egg. However, we noticed that in species that acquire the linear-type arrangement, such as *Zeldia*, cells at the two-cell stage are likely to divide perpendicularly to each other (Schulze and Schierenberg, 2011). As this species has a slender eggshell (AR=2.5, Table S1), we suspected that the eggshell shape induced the linear-type arrangement. In summary, we suspect that the shape of eggshell is a major parameter determining the pattern of cell arrangement, and is responsible for the generation of diversity therein.

### Deformation of eggshell shape in *C. elegans* mutants and RNAi-treated strains

**Fig. 2.**
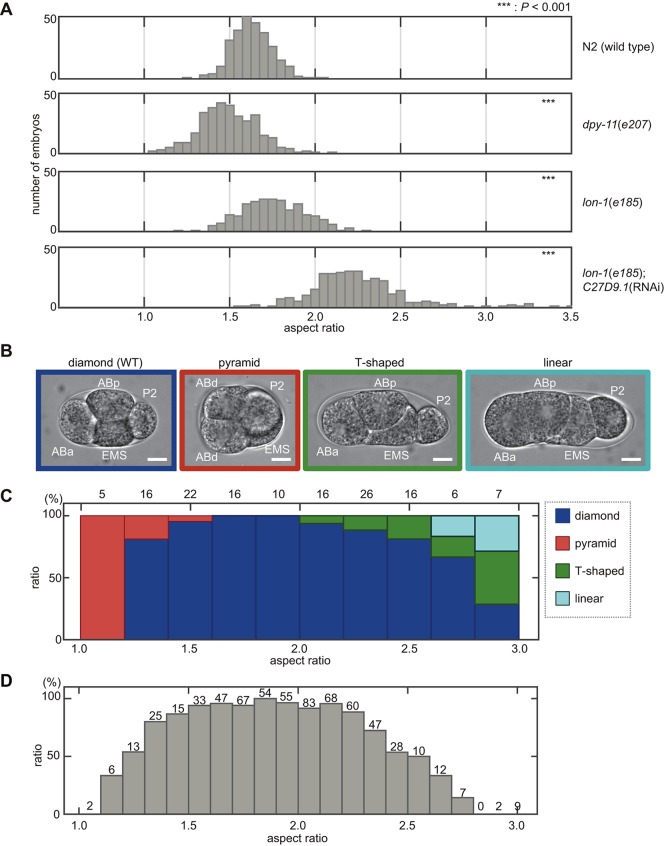
**Eggshell shape determines cell arrangement pattern in the *C. elegans* embryo.** (A) Histograms showing the ARs in *C. elegans* mutants and RNAi-treated strains. The means±s.d. of the eggshell shapes from all embryonic stages are: N2 (wild type) (1.6±0.1, *n*=281), *dpy-11*(*e207*) (1.5±0.2, *n*=331), *lon-1*(*e185*) (1.8±0.2, *n*=258), *lon-1*(*e185*); *C27D9.1* (RNAi) (2.3±0.3, *n*=322). ***P<0.001 versus N2 (wild type). Student's *t*-test was used for *dpy-11*(*e207*), *lon-1*(*e185*); Wilcoxon's rank-sum test was used for *lon-1*(*e185*); *C27D9.1* (RNAi). (B) Micrographs showing the different cell arrangement patterns at the four-cell stage of *C. elegans* embryos: for the pyramid-type arrangement, the daughter cells of the AB cell are indicated ‘ABd’ as we were unable to distinguish between ABa and ABp in this arrangement. Scale bars: 10 μm. (C) Relationship between the percentage of the four types of cell arrangement found (blue, diamond type; red, pyramid type; green, T-shaped type; cyan, linear type) and the ARs (*n*=188). The data are for four strains: N2, *dpy-11*(*e207*), *lon-1*(*e185*), and *C27D9.1* RNAi-treated strains on a *lon-1*(*e185*) background. The numbers above the bars represent the number of the four-cell stage embryos. (D) Dependence of hatch rate on AR (*n*=643); the data are for five strains: N2, *dpy-11*(*e207*), *lon-1*(*e185*) and *C27D9.1* RNAi-treated strains on a N2 or *lon-1*(*e185*) background. The numbers above the bars represent the number of embryos.

To test whether deformation of the eggshell shape affects the cell arrangement pattern, we searched for genes involved in the determination of eggshell shape in *C. elegans*. In the WormBase database (www.wormbase.org), phenotypes of six mutants were categorized as ‘egg round’ or ‘egg long’. As we aimed to change the eggshell shape without affecting other processes in embryogenesis as much as possible, we excluded four mutants (*emb-18*, *emb-21*, *emb-25* and *emb-30* (also known as *apc-4*) that have an embryonic lethal phenotype. The ARs of the *ceh-18*(*mg57*) mutant were not significantly different from those of the wild type in our analyses (Fig. S1B). Thus, the only remaining mutant was the *lon-1*(*e185*) mutant. The ARs of the mutant embryos were significantly larger than those of the wild type (Fig. 2A). LON-1 shares homology with the cysteine-rich secretory protein (CRISP) family of proteins, is a downstream target of the TGF-β signaling pathway, and is expressed mainly in the hypodermis (Maduzia et al., 2002; Morita et al., 2002). A previous study in our laboratory (Hara and Kimura, 2009) implied that RNAi-mediated knockdown of *C27D9.1* increases the AR. *C27D9.1* is an uncharacterized gene whose product has a domain homologous to fucosyltransferase. Here, we confirmed that RNAi of this gene increased the AR (Fig. S1B). Furthermore, we succeeded in obtaining high-AR embryos by knocking down *C27D9.1* in a *lon-1*(*e185*) background (Fig. 2A).

Next, we attempted to obtain embryos with ARs lower than those of the wild type. We successfully obtained long (high-AR) embryos from the *lon-1* mutant strain, whose adult body shape is also long (Maduzia et al., 2002; Morita et al., 2002); therefore, we speculated that it is possible to obtain short (low-AR) embryos from short adults (Fig. S1C). We examined a mutant strain with short adults, *dpy-11*(*e207*) (Ko and Chow, 2002), and found that it produced low-AR embryos (Fig. 2A). *dpy-11* encodes a membrane-associated thioredoxin-like protein expressed exclusively in the hypodermis (Ko and Chow, 2002). In summary, we successfully obtain *C. elegans* embryos with ARs ranging from 1.0 to 3.5. The eggshell shape was axially symmetric along the long (AP) axis (Fig. S1D).

### Patterns of cell arrangements are altered in eggshell shape variants of *C. elegans* embryos

To evaluate the effect of eggshell shape on the pattern of cell arrangement, we observed cell arrangements at the four-cell stage for the different eggshell shapes. Normally, at the four-cell stage, *C. elegans* embryos acquire the diamond-type of cell arrangement, in which the nuclei are positioned at the vertexes of a diamond shape (Fig. S1A). The embryonic cells of *C. elegans* are named after the mother cell and their position relative to the sister cells (Sulston et al., 1983). At the four-cell stage, all cells (ABa, ABp, EMS and P2) except ABa and P2 make contact with each other (Fig. 2B; Fig. S1A).

We succeeded in altering cell arrangements by changing the eggshell shapes (Fig. 2B). When the AR decreased to below 1.5 (i.e. the eggshell was more circular), the pyramid-type of cell arrangement was observed, in which all four cells, including ABa and P2, made contact with each other (Fig. 2B; Fig. S2B). The pyramid-type of cell arrangement was dominant when the AR was below 1.2 (Fig. 2C). In contrast, when the AR exceeded 2.0, the T-shaped type of cell arrangement appeared, in which neither ABp and P2 nor ABa and P2 made contact (Fig. 2B; Fig. S2B). The T-shaped type was the most frequently observed when the AR exceeded 2.8 (Fig. 2C). When the AR exceeded 2.7, the linear type appeared, in which the four nuclei are arranged linearly: ABa and EMS, ABa and P2, and ABp and P2 do not contact each other (Fig. 2B,C; Fig. S2B). These changes in the pattern of cell arrangement are not considered a direct consequence of mutation or knockdown of the targeted genes because (1) the patterns varied, even among embryos of the same genotype, in an AR-dependent manner (Fig. S2B), (2) both the change in the patterns and ARs in *dpy-11* and *C27D9.1* single dysfunction were suppressed by *dpy-11; C27D9.1* double dysfunction (Fig. S2B), and (3) the genes involved in deformation of eggshell shape (i.e. *lon-1*, *C27D9.1*, and *dpy-11*) were non-essential genes that did not affect the intrinsic orientation of cell division (Figs S1A and S2A).

Overall, these results demonstrate that the pattern of cell arrangement can be altered by changing the eggshell shape. The hypothesis that eggshell shape contributes to diversity in cell arrangements was supported by the fact that the various patterns of cell arrangement observed in different nematode species (Fig. 1C) could be reproduced in *C. elegans* embryos with differing eggshell shapes (Fig. 2C).

### Robustness of the diamond-type cell arrangement in the *C. elegans* embryo

On changing the eggshell shape, the diamond type was observed to predominate in a wide range of ARs (from 1.2 to 2.8) (Fig. 2C). This range includes that observed for wild-type eggshells, in which ARs vary from 1.3 to 2.0 (Fig. 2A), and is consistent with the range of ARs in other nematode species displaying the diamond-type arrangement (Fig. 1C). We demonstrated robustness by mechanically deforming the eggshell of the wild-type embryos (Fig. S2C). By embedding embryos into microchambers (Minc et al., 2011), we obtained embryos with ARs from 1.4 to 2.5. The embryos retained normal arrangement within this range, further supporting the robustness of the arrangement.

To correlate the robustness of the diamond-type arrangement with the robustness of embryogenesis, we quantified the hatching rate of embryos with different eggshell shapes. The hatching rate represents the proportion of embryos that hatch from the eggshell to become L1 larvae, implying normal embryogenesis. The hatching rate decreased with increasing deviance of the AR from that of the wild type (Fig. 2D; Fig. S2D), and was consistent with the proportion of embryos acquiring the diamond-type arrangement (Fig. 2C). The failure of embryogenesis at low ARs, which is consistent with what has been previously reported for the *spv-1* mutant (Tan and Zaidel-Bar, 2015), indicates that the pyramid-type arrangement (abnormal contact between ABa and P2 cells) has a deleterious effect on embryogenesis. The failure of embryogenesis at high ARs is consistent with the notion that contact between ABp and P2 cells is important for dorsal-ventral axis formation (Gönczy and Rose, 2005; Priess, 2005). We confirmed that embryos acquiring a pattern of cell arrangement other than the diamond type did not develop to hatching. Overall, the results support the notion that the robustness of the diamond-type cell arrangement is critical for the robustness of embryogenesis against eggshell deformation.

### Computer simulation of the repulsion-only model

**Fig. 3.**
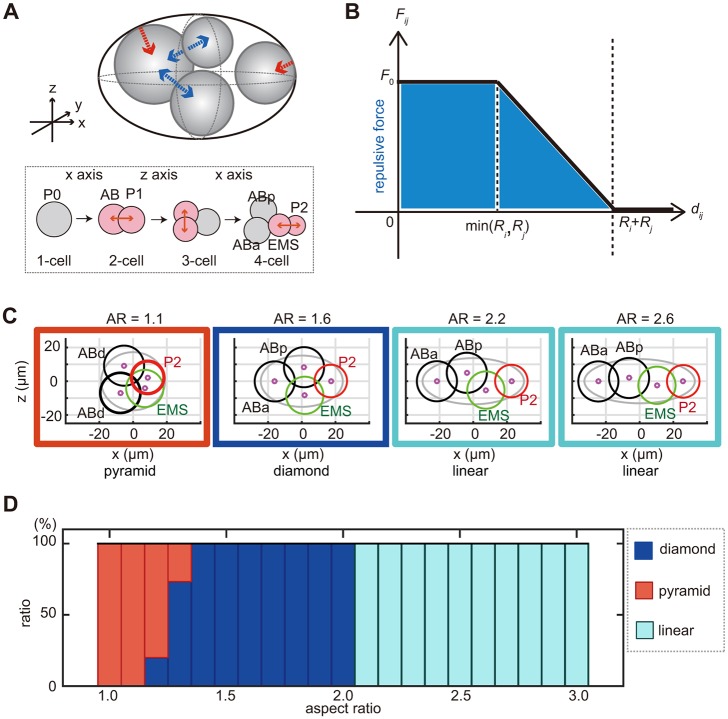
**Simulation of cell arrangement patterns with the RO-only model.** (A) Schematic representation of the RO model. The upper panel shows the repulsive forces between the cells (blue arrows) and between the cells and the eggshell (red arrow). The lower panel shows the orientation and timing of cell divisions. (B) Relationship between strength of intercellular force (*F_ij_*) and cell–cell distance (*d_ij_*) in the RO model. (C) Examples of the cell arrangement patterns at the four-cell stage as simulated by the RO model at AR=1.1, 1.6, 2.2 and 2.6, respectively. The black circle represents AB daughter cells; the green, the EMS cell; and the red, the P2 cell. The magenta circle represents the centroid of the cells. (D) Relationship between the percentage of different patterns of cell arrangements found and the ARs in the RO model (blue, diamond type; red, pyramid type; cyan, linear type).

In order to elucidate the mechanical basis of the cell arrangements, we investigated whether an existing mechanical model of cell arrangement accounts for diversity and robustness against deformation of the eggshell. The model of Fickentscher et al. (2013) accurately accounts for the arrangement and trajectory of cells from the two-cell to 12-cell stages of *C. elegans* embryos with normal eggshell shape (i.e. AR=1.7; Fickentscher et al., 2013), under the condition that the direction, timing and volume ratio of cell division are provided. The model considers two types of repulsive forces: between neighboring cells and between a cell and the eggshell (Fig. 3A). In this report, we termed this model the repulsion-only (RO) model. In this model, the strength of the repulsive force depends on the distance between the centers of two cells (Fig. 3B) or between the cell center and the nearest part of the eggshell. Here, the cell center is defined as the center of the nucleus.

We examined whether the RO model also reproduces diverse patterns of cell arrangements when eggshell shape is changed while other parameters are maintained. The diamond-type arrangement was observed in 100% of simulations with ARs from 1.4 to 2.0 (Fig. 3C,D). Interestingly, when the AR exceeded 2.0, the diamond type was not observed, and 100% of simulations resulted in the linear-type arrangement (Fig. 3C,D). This did not reflect the situation *in vivo*, where the proportion of embryos with the diamond type decreased gradually when the AR exceeded 2.0, and more than 50% of the embryos showed the diamond type even for ARs from 2.6 to 2.8 (Fig. 2C). Therefore, the RO model was less robust against eggshell deformation than real embryos. Another notable difference between the real embryos and the RO model was that the model did not reproduce the T-shaped arrangement at any AR (Fig. 3D); in contrast, in real embryos, this type of arrangement was observed for ARs over 2.0 (Fig. 2C). Instead, the linear type was obtained 100% of the time in the RO model for ARs over 2.0 (Fig. 3D), whereas this type was observed in the real embryos only when the AR exceeded 2.7 (Fig. 2C). We confirmed that changing other parameters in this model did not affect the ability of this model to reproduce diversity and robustness (Fig. S3). From these results, we conclude that the RO model is not sufficient to explain the diversity and robustness of cell arrangements against eggshell deformation.

### Characterization of the intercellular forces in the *C. elegans* embryo

**Fig. 4.**
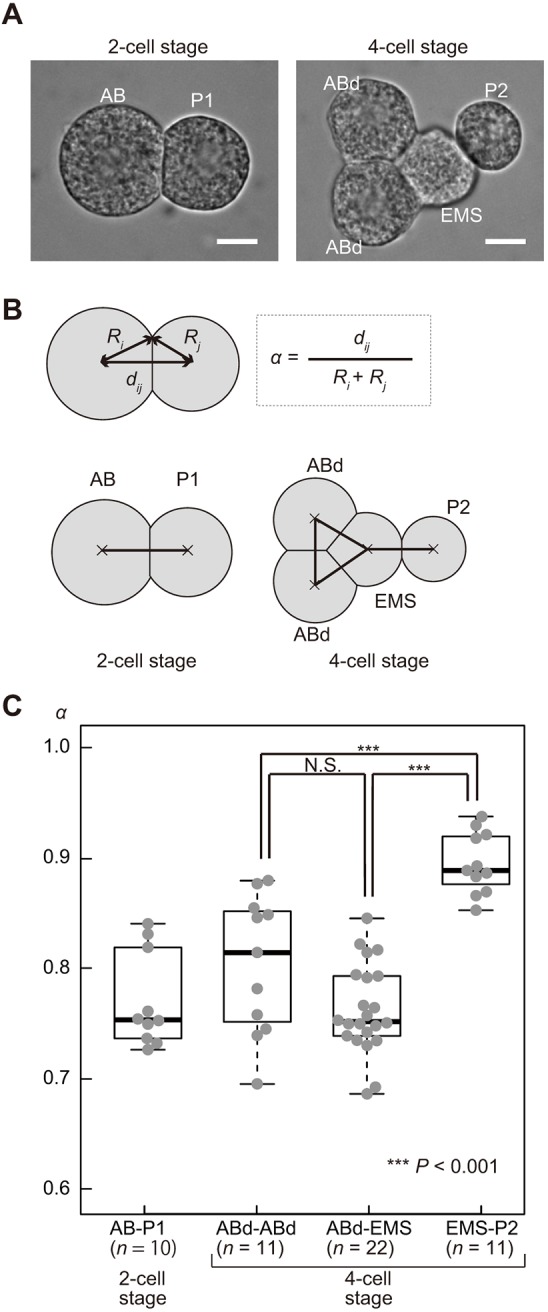
**Asymmetric attraction forces between blastomeres at the four-cell stage in the *C. elegans* embryo.** (A) Micrographs showing *C. elegans* embryos at the two- or four-cell stage, with the eggshell removed. The daughter cells of the AB cell were marked as ‘ABd’ as we were unable to distinguish ABa and ABp cells when the eggshell was removed. Scale bars: 10 μm. (B) Definition of the stable repulsion ratio (*α*), which is calculated as the distance between cells (*d_ij_*) divided by the sum of radii (*R_i_*+*R_j_*) of the cells. The diagram shows the combinations of the cells used to measure cell–cell distances at the two- or four-cell stage. (C) Bee swarm plot and box plot of *α* for each combination of cell types [AB and P1 cells (two-cell stage, *n*=10), AB daughter cells (four-cell stage, *n*=11), AB daughter and EMS cells (four-cell stage, *n*=22), and EMS and P2 cells (four-cell stage, *n*=22)]. The box and whiskers are drawn as in Fig. 1C. ***P<0.001, Student's *t*-test.

In order to elucidate the mechanisms underlying the diversity and robustness of cell arrangements against eggshell deformation, we observed the physical interactions between embryonic cells upon the eggshell removal. We noticed that, even when cells were not confined within the eggshell, divided cells did not repel each other completely, but remained attached to each other to some extent at the two-cell and four-cell stages (Fig. 4A). We concluded that, in addition to the repulsive forces, attractive forces exist. We defined a parameter termed the ‘stable repulsion ratio (*α*)’ as the distance between the centers of the two cells (*d_ij_*) divided by the sum of the radii of the two cells (*R_i_+R_j_*) under the condition where the eggshell is removed (Fig. 4B). When there are only repulsive forces, *α* should be 1.0, as the two cells repulse each other until they are completely separated. For real embryonic cells, *α* was less than 1.0 (Fig. 4C). The results indicated that, when the distance between the centers of the cells is short [*d_ij_*<*α*(*R_i_*+*R_j_*)], the repulsive force is dominant; however, when the distance increases [*d_ij_*>*α*(*R_i_*+*R_j_*)], the attractive force is dominant, so that the distance between the centers of the cells stabilizes at an intermediate distance [*d_ij_*=*α*(*R_i_*+*R_j_*)].

Moreover, we found that the degree of attractive force was not uniform but rather was asymmetric, depending on the cell type. At the four-cell stage, ABa, ABp and EMS cells were tightly attached (Fig. 4A), and *α* was ∼0.75 (Fig. 4C). In contrast, P2 was loosely attached to EMS (Fig. 4A), and *α* for the EMS–P2 contact was ∼0.90 (Fig. 4C). These results indicate that, in addition to the repulsive forces, asymmetric attractive forces are present between the cells at the four-cell stage of *C. elegans* embryos.

### Computer simulation of the asymmetric attraction model

**Fig. 5.**
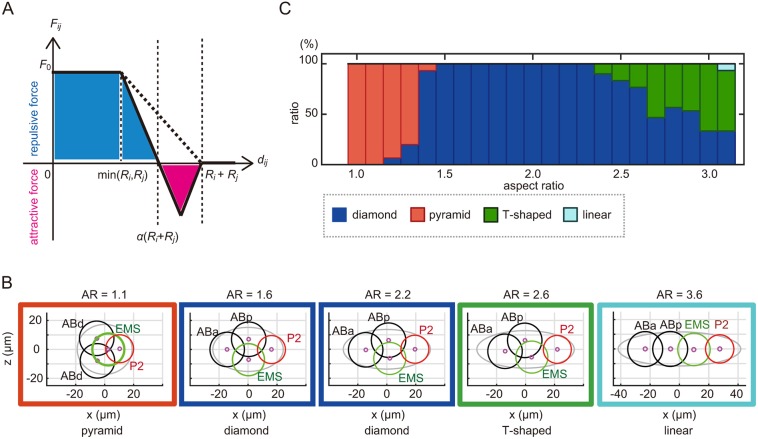
**Simulation of cell arrangement patterns with the AA model.** (A) Relationship between the strength of intercellular forces (*F_ij_*) and cell–cell distance (*d_ij_*) in the AA model. (B) Examples of the cell arrangement patterns at the four-cell stage as simulated by the AA model at AR=1.1, 1.6, 2.2, 2.6 and 3.6, respectively, as in Fig. 3C. (C) Relationship between the percentage of different types of cell arrangement found (blue, diamond type; red, pyramid type; green, T-shaped type; cyan, linear type) and the ARs in a computer simulation based on the AA model [*α*=0.90 (EMS and P2), and *α*=0.75 (others)].

To test whether the asymmetric attractive forces represent the hitherto unknown mechanism underlying the diversity and robustness of cell arrangements, we revised the RO model by adding the asymmetric attractive forces between the cells, as observed *in vivo*. We termed the revised model as ‘the asymmetric attraction (AA) model’. Under the AA model, we assumed that the intercellular force (*F_ij_*) becomes zero at an intermediate distance, when the distance between the centers of the two cells (*d_ij_*) is *α*(*R_i_*+*R_j_*) (Fig. 5A). When *d_ij_*>*α*(*R_i_*+*R_j_*), the attractive force acts between the centers of the two cells for as long as they are attached (*d_ij_*<*R_i_+R_j_*) (Fig. 5A), and the distance between the cells decreases until it reaches *d_ij_*=*α*(*R_i_*+*R_j_*). We set *α* to 0.90 for the interaction between EMS and P2 cells, and to 0.75 for other interactions, based on the experimental measurements (Fig. 4C).

The behaviors predicted by the AA model resembled those of real embryos. First, the AA model reproduced the diamond type of cell arrangement when the AR was 1.6 (Fig. 5B), which is the average AR for wild-type embryos measured in this study. Second, the model reproduced the pyramid-type arrangement when the AR was 1.1 (Fig. 5B). These two features were also observed in the RO model (Fig. 3C). Third, and most importantly, the diamond-type arrangement was more robust against changes in the AR in the AA model than in the RO model. The AA model produced the diamond-type pattern even when the AR exceeded 3.0 (Fig. 5C), whereas the RO model did not produce the diamond-type pattern when the AR exceeded 2.0. Fourth, the T-shaped type, which was observed *in vivo* for high ARs but was not reproduced in the RO model, was reproduced in the AA model (Fig. 5B,C). The T-shaped type is defined by the loss of contact between ABp and P2, compared with the diamond type. Fifth, the AA model predicted the linear type for ARs exceeding 3.1 (Fig. 5B,C). In summary, the model that considered the asymmetric attractive forces increased the robustness of the diamond-type cell arrangement and successfully reproduced all types of cell arrangement patterns observed in real embryos, including those in nematode species other than *C. elegans*. The modeling demonstrated that the framework of the AA model is sufficient to produce the diversity and the robustness of cell arrangement.

### Attractive forces are produced by E-cadherin in the *C. elegans* embryo

Adhesion between cells may be explained by the difference in interfacial tension between cells and between cells and the medium, as observed in soap bubbles that are in contact (Hayashi and Carthew, 2004; Pierre et al., 2016). Cadherin proteins (Yoshida and Takeichi, 1982) are cell surface proteins that promote cell adhesion and thus provide attractive forces by interacting with each other on the surface of the other cell (termed ‘*trans*-interaction’). Cadherins reduce interfacial tension by decreasing cortical tension and increasing adhesive tension between cells (Lecuit and Lenne, 2007; Maître and Heisenberg, 2013; Winklbauer, 2015). The *trans*-interaction of cadherins is dependent on Ca^2+^ (Yoshida and Takeichi, 1982). We demonstrated that the attraction between the *C. elegans* blastomeres was reduced under Ca^2+^-free conditions (Fig. S4), suggesting that the attractive forces were dependent on cadherins in the present system.

**Fig. 6.**
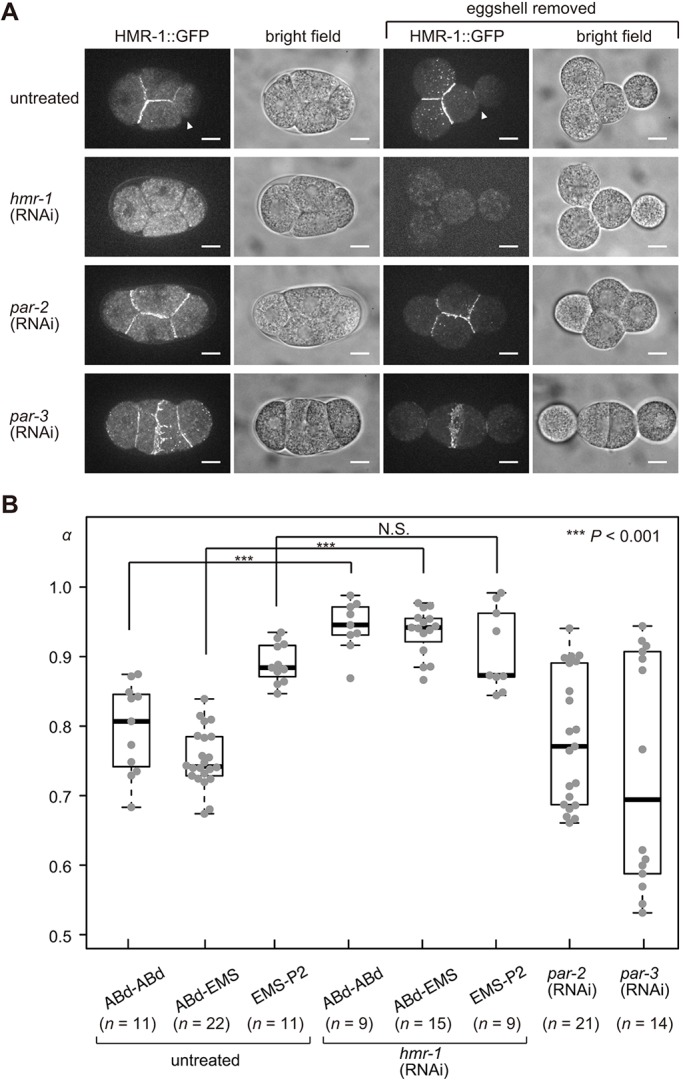
**Cadherin localization and cell adhesion at the four-cell-stage in the *C. elegans* embryo.** (A) Micrographs of embryos expressing HMR-1 fused to GFP protein (LP172 strain) in untreated, *hmr-1* (RNAi), *par-2* (RNAi) and *par-3* (RNAi) embryos, with or without eggshells. White arrowheads indicate cell–cell contact between EMS and P2 cells. Note that, for *par-2* (RNAi) and *par-3* (RNAi) embryos, the cell division orientations and cell arrangement patterns are variable and the micrographs presented here are examples (see also Fig. 8 for further examples showing the variability). Scale bars: 10 μm. (B) *α* of untreated, *hmr-1* (RNAi), *par-2* (RNAi) and *par-3* (RNAi) embryos without eggshells at the four-cell stage. The box and whiskers are drawn as in Fig. 1C. ***P<0.001, Welch's *t*-test for ABd-ABd pair, ABd-EMS pair, and EMS-P2 pair.

*hmr-1* encodes E-cadherin in *C. elegans*, and it has been shown that the HMR-1 protein localizes at cell adhesion sites from the embryonic stage onward (Grana et al., 2010; Pettitt, 2005). We found that, at the four-cell stage, HMR-1 accumulation was asymmetric as there was less HMR-1::GFP intensity at the EMS–P2 contact in HMR-1::GFP-expressing embryos (Fig. 6A), consistent with the asymmetric attraction model (Fig. 4). Because HMR-1 asymmetrically localizes to the anterior cortex at the one-cell stage in a manner dependent on the partitioning-defective (*par*) gene family (Munro et al., 2004), we examined HMR-1 localization in embryos where *par*-2 or *par-3* has been knocked down by use of RNAi (Fig. 6A). The asymmetry of HMR-1 localization along the anterior-posterior axis was lost in *par*-knockdown cells, as expected. Interestingly, however, the HMR-1 localization was not uniform but variable among the cell contacts. In agreement with the non-uniform HMR-1 localization, the *α* parameter in *par*-knockdown cells was also variable (Fig. 6B). In conclusion, the asymmetric attractive forces that are weak at the EMS–P2 contact might be explained by the weak HMR-1 localization observed at this contact, and this localization is regulated through cell polarity established by the *par* genes.

To test whether HMR-1 is required for the attractive forces, we knocked down *hmr-1* by RNAi. When the eggshell was removed, cell–cell adhesion was impaired, resulting in cells of nearly spherical shape (Fig. 6A). To quantify the degree of loss of adhesion, we measured the *α* parameter for *hmr-1*-knockdown embryos. The parameter was significantly larger for *hmr-1* knockdown embryos than for wild-type embryos (Fig. 6B). This indicated that the E-cadherin HMR-1 is necessary to produce the attraction forces at the four-cell stage.

### Attraction forces are necessary to produce the robustness of the diamond-type cell arrangement

The present physical modeling study (Fig. 5) suggested that attractive forces contribute to the robustness of the diamond-type arrangement against eggshell deformation. The model predicted that, without attractive forces, the diamond-type arrangement would be lost at high ARs (Fig. 3D). To test this prediction, we attempted to experimentally reduce the attractive forces. In an initial attempt, we knocked down *hmr-1* alone in a *lon-1*(*e185*) background. However, the diamond-type arrangement was still observed in embryos with high ARs over 2.0 (Fig. S5A).

**Fig. 7.**
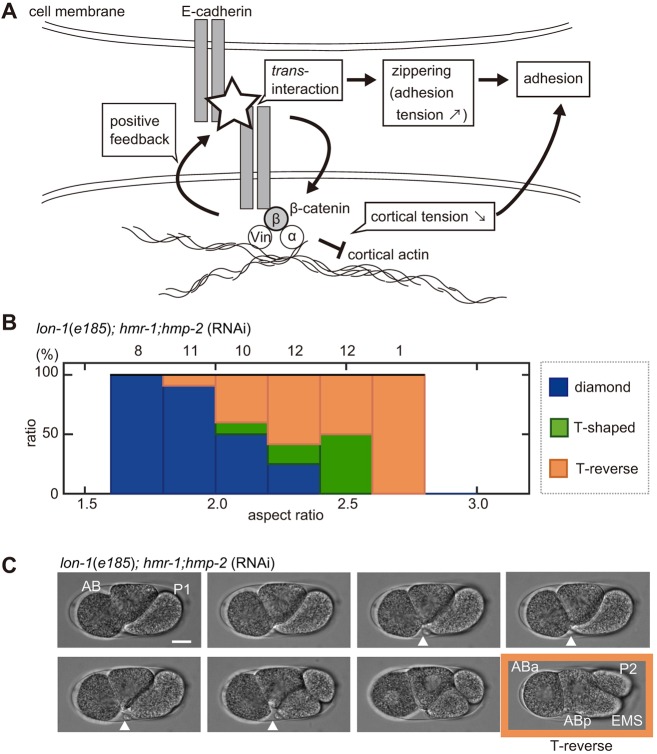
**Impaired robustness of the diamond-type arrangement against eggshell deformation in the *C. elegans* embryo.** (A) Schematic representation of E-cadherin- and β-catenin-mediated cell adhesion at cell boundaries. α-catenin (α) and Vinculin (Vin) mediate interactions with the cortical actin. (B) Relationship between the percentage of the patterns of cell arrangements found (blue, diamond type; green, T-shaped type; orange, T-reverse type) and the ARs in the *hmr-1*; *hmp-2*-knockdown strain on a *lon-1(e185)* background; the numbers above the bars represent the number of embryos. (C) Sequential snapshots acquired when breakage of cell adhesion between ABa and EMS cells occurred in the *hmr-1; hmp-2*-double-knockdown strain with *lon-1*(*e185*) mutant background. White arrowheads indicate cell–cell contact between ABa and future EMS cell. The T-reverse-type cell arrangement (absence of cell–cell contact between ABa and P2 cells, and ABa and EMS cells) was formed at the four-cell stage (orange). Scale bar: 10 μm.

To further impair the attractive forces, we knocked down β-catenin (*hmp-2*), which mediates the cadherin–actin interaction (Fig. 7A), in addition to E-cadherin (*hmr-1*). First, we confirmed that *hmr-1;hmp-2* embryos acquired the diamond-type arrangement in the normal range of ARs (1.6–2.0) (Fig. 7B, Fig. S5A). Importantly, the majority of *hmr-1;hmp-2* embryos lost the diamond-type arrangement at ARs over 2.0 (Fig. 7B, Fig. S5A). The experimental results agreed with the prediction from the model without attractive forces (i.e. the RO model); according to this model, without attractive forces, the range of ARs that supported the diamond-type was narrower and, thus, the arrangement was less robust (Fig. 3D). It should be noted that the model simulation predicted that the linear type of cell arrangement would appear; however, the majority of real embryos adopted a T-reverse-type shape at high ARs (Fig. 7B), in which ABp was in contact with EMS and P2, while ABa and EMS were not in contact (Fig. 7C) (see Discussion for possible explanations). In summary, a reduction in robustness is observed upon impairing the attractive forces through *hmp-2* and *hmr-1* knockdown.

### The AA model recapitulates the effect of changing the orientation of cell division

**Fig. 8.**
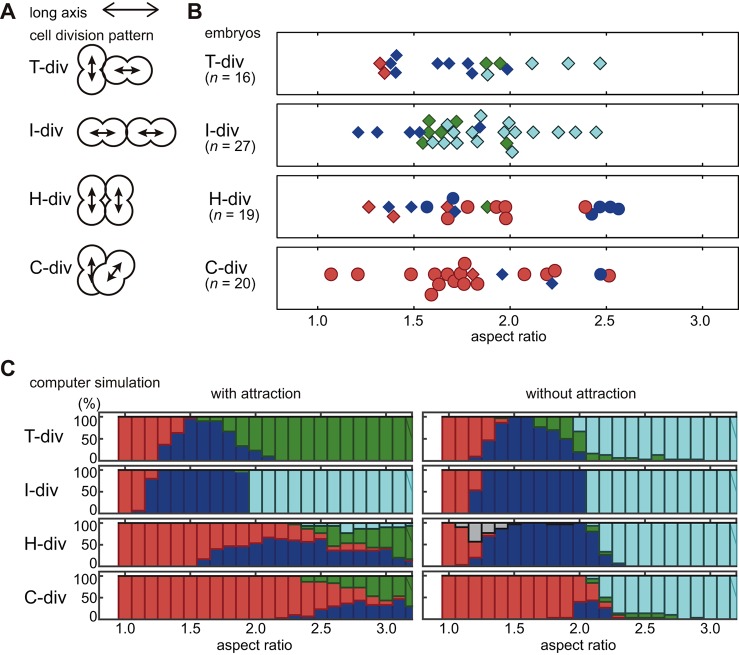
**Variation of cell arrangement patterns by orientation of cell division.** (A) Classification of cell division patterns by orientation of cell divisions; T-div, one cell divides perpendicularly to the long axis, the other cell divides parallely along the long axis; I-div, both cells divide parallely to the long axis; H-div, both cells divide perpendicularly to the long axis and parallely to each other; C-div, both cells divide perpendicularly to the long axis, perpendicularly to each other and not on the same plane. (B) Cell arrangement patterns for each cell division orientation observed in *par-2* (RNAi) (circle) or *par-3* (RNAi) (diamond) embryos plotted against AR (red, pyramid; blue, diamond; green, T-shaped; cyan, linear). (C) Relationship between percentage of different patterns of cell arrangements found and the ARs in the AA model with attraction (left) or without attraction (i.e. the RO model, right) depending on the cell division orientation and ARs. Blue, diamond type; red, pyramid type; green, T-shaped type; cyan, linear type; gray, H-shaped type in which all cells are only in contact with two neighboring cells.

So far, we have focused on explaining the effect of changing the eggshell shape when the orientation of cell division is fixed as being the same as that in wild-type embryos (‘T-div’ in Fig. 8A). The orientation of cell division affects the cell arrangement (Akiyama et al., 2010; Pierre et al., 2016). We aimed to determine whether the AA model is applicable to cases with different orientations of cell division. We investigated *par-2* (RNAi) embryos, in which the two cells tend to divide perpendicularly to the AP axis (Fig. 8A, ‘H-div’ and ‘C-div’), and *par-3* (RNAi) embryos, in which both cells tend to divide along the AP axis (Fig. 8A, ‘I-div’) (Kemphues et al., 1988).

We characterized the initial orientation of cell division at the two-cell stage and correlated it with the cell arrangement at the four-cell stage (Fig. 8A,B). In embryos where the two cells initially divide along the long axis of the embryo (termed ‘I-div’ because the arrangement looks like the character I), which are observed under the *par-3* (RNAi) condition, the cells tend to adopt the linear arrangement when the AR is high (>1.6). However, the diamond or T-shaped arrangements were observed at lower ARs (Fig. 8B). ‘H-div’ refers to the arrangement in which the division axes are parallel, as this arrangement resembles the character H; ‘C-div’ represents the arrangement in which the division axes are perpendicular (cross configuration) to each other. In H-div and C-div embryos, which are observed mainly under the *par-2* (RNAi) condition, the cells tend to acquire the pyramid arrangement at a wide range of ARs (Fig. 8B). Interestingly, in about half of the *par-3* (RNAi) embryos, one of the two cells initially divided perpendicularly to the long axis whereas the other divided along the long axis (‘T-div’, as the arrangement resembles the character T). This initial orientation of cell division is the same as in the wild type. Nevertheless, the diamond-arrangement was not dominant in T-div embryos at the range of ARs (2.0–2.5) at which this arrangement is generally dominant in the wild type (Fig. 2C).

Next, we conducted simulations with the altered orientations of cell division (Fig. 8C). For input parameters, we used *α*=0.75, which was close to the mean of the experimental values of *par-2* (RNAi) and *par-3* (RNAi) embryos (Fig. 6B). We additionally changed the timing of cell division so that the AB and P1 divisions were simultaneous, and the volume of the four daughter cells were equivalent, because *par-2* (RNAi) and *par-3* (RNAi) impairs the asymmetry in division timing and cell volume (Kemphues et al., 1988). The AA model recaptured most features of the experimental observations, as follows (Fig. 8C). Under the I-div condition, the linear arrangement was dominant for AR≥2.0. Under the H-div and C-div conditions, the pyramid arrangement was dominant over a wide range of ARs. Under the T-div condition, the AA model recaptured the reduced robustness of the diamond arrangement, as observed *in vivo*. This implies that the difference in cell division timing and/or asymmetry in cell volume was important for the robustness of the diamond arrangement. There was a discrepancy between the AA model and experimental results in that, under the T-div condition with AR≥1.8, the linear arrangement was dominant *in vivo* (Fig. 8B), whereas the T-shaped arrangement was dominant in the AA model (Fig. 8C). When we conducted RO model simulation (i.e. no attraction in the AA model), the linear arrangement was captured at AR≥2.0 under the T-div [*par-3* (RNAi)] condition (Fig. 8D). The RO model additionally recaptured the I-div [*par-3* (RNAi)] condition, but not the H-div or C-div (*par-2* RNAi) conditions (Fig. 8D). As the RO model better recapitulates the features of the T-div (*par-3* RNAi) condition, the AA model with adjusted strength of attractive forces should agree with the *par-3* (RNAi) condition. The strength of the attractive forces was highly variable under the *par-3* (RNAi) condition (Fig. 6B). Therefore, an understanding of the mechanism underlying the regulation of attractive forces under *par-3* (RNAi) conditions is needed in order to develop a model that accounts for this condition. The results collectively support that the AA model presented is suitable for examining the effect of cell division orientation as well as the effect of eggshell shape on the determination of the cell arrangement pattern.

## DISCUSSION

### Diverse patterns of cell arrangements in nematode embryos in the four-cell stage

The cell arrangement patterns at the four-cell stage of various nematode species can be classified into four different types, which correlate with the shape (i.e. the AR) of the eggshells. In this study, we succeeded in changing the diamond-type cell arrangement of *C. elegans* embryos into the other three types, demonstrating that eggshell shape is sufficient to change the cell arrangement pattern. The AR dependency of these patterns in various nematode species resembled that in *C. elegans*. Because our AA model accounts for the AR dependence in *C. elegans*, it well explains the diversity in cell arrangement patterns in various nematode species.

The AR dependence of the cell arrangement patterns in various nematode species was not the same as that in *C. elegans*. An apparent difference is that some nematode species with ARs of 2.0–2.6 adopt a linear-type arrangement (Fig. 1C); however, *C. elegans* embryos with ARs in this range do not (Fig. 2C). The arrangements in these species can be explained with the AA model. In the absence of attractive forces, the AA model coincides with the RO model (Fickentscher et al., 2013) by definition. The RO model predicts the linear-type arrangement when the AR exceeds 2.0 (Fig. 3D). Therefore, nematode species that adopt the linear-type arrangement with ARs of 2.0–2.6 are explained by the AA model with reduced attractive forces.

### Changing eggshell shape through gene manipulation

In this study, we obtained eggshells with various ARs by using gene manipulation. We found a correlation between the thickness of the adult body and the roundness of the egg (Fig. 2, Fig. S1). We speculated that adults with thicker bodies would have thicker gonads, and eggs passing the thicker gonads would be rounder (Fig. S1C). This idea is consistent with the observation that increasing the volume of the egg via *C27D9.1* RNAi resulted in the formation of slender eggs, possibly because the gonad of this mutant is relatively thin compared to the enlarged egg (Fig. S1). In future studies, it would be of interest to investigate the correlation between the thickness of the gonad and the shape of the eggs by examining various nematode species (Goldstein, 2001; Schulze and Schierenberg, 2011).

### Roles of E-cadherin and β-catenin in the attractive forces

We showed that attractive forces between cells are important for the diversity and robustness of cell arrangements. In our measurement of the *α* parameter in embryos without eggshell, the knockdown of *hmr-1* or *hmp-2* alone severely impaired the attractive forces (Fig. 6B, Fig. S5C). However, knockdown of the both genes simultaneously was required for loss of robustness in embryos inside the eggshell. We speculate that compression by the eggshell increases the attachment between cells and induces passive *trans*-interaction of E-cadherins, thus increasing the attractive forces in a β-catenin-dependent manner. This may be consistent with the positive-feedback regulation (Leckband and de Rooij, 2014) or the adhesion coupling (Maître and Heisenberg, 2013) between cadherins and cortical actin mediated by catenins. However, we cannot exclude the possibility that *hmr-1;hmp-2* RNAi caused an uncharacterized defect, independently of attractive forces, which affected the robustness of the diamond-type arrangement. Cadherin- and catenin-mediated adhesions between the cells are important for cell sorting and migration during development (Leckband and de Rooij, 2014). The present study demonstrates an additional role for cell adhesion in the establishment of diversity and robustness of cell arrangements.

### Limitations of the AA model

One apparent discrepancy between the cell arrangements in the *C. elegans* embryos and our model is that, without the attractive forces in the AA model (i.e. RO model), the linear type of cell arrangement was expected for longer embryos. However, embryos were found to adopt a T-reverse-type arrangement under *hmr-1*; *hmp-2* RNAi (Figs 3D, 7B,C). There are two possible reasons for the discrepancy. First, the cell shape is believed to affect the orientation of the cell division axis. In slender eggshells, cells elongate along the long axis at the two-cell stage. The cell division axis of the elongated AB cell is considered to tilt toward the long axis as this cell tends to divide along its long axis (Hertwig's rule) (Minc et al., 2011). Second, the AA model assumes the eggshell to be ellipsoidal, but in fact eggshells with high ARs were observed to be thicker near the poles. EMS and P2 cells were arranged perpendicular to the long axis in the thick region, with enough space to position cells according to a T-reverse type of cell arrangement.

### The AA model as a framework to explain cell arrangement by incorporating the effect of spatial constraints and cell division orientation

Cell arrangement patterns are determined by the coordination of cell division orientation, position and timing, in addition to physical interaction between cells and the spatial confinement of the eggshell. The present model is capable of examining the effects of changing these parameters: here, we recapitulated the effect of changing the orientation of cell division, as observed in *par-2* and *par-3* mutants (Fig. 8). In addition to the interplay between cell division orientation, and cell and eggshell shape, cell arrangement patterns are determined by the strength of attractive and repulsive forces. The present AA model provides a good framework for integration of these parameters into a single model.

## MATERIALS AND METHODS

### *C. elegans* strains

The *C. elegans* strains used in this study are listed in Table S2. *C. elegans* strains and *Diploscapter coronata* were maintained using a standard procedure for *C. elegans* (Brenner, 1974; Stiernagle, 2006). *Aphelenchoides besseyi* was maintained on potato dextrose agar (PDA) plates cultured with *Botryllus cinera* (Yoshida et al., 2009).

### RNAi

Genetic knockdown of *C27D9.1*, *par-2*, *par-3*, *hmr-1*, and *hmp-2* was established by feeding RNAi, as described previously (Kamath et al., 2000). For *hmr-1* RNAi, a probe targeting base pairs 13,610 to 14,254 of the unspliced *hmr-1* gene was amplified from genomic DNA and cloned into a L4440 plasmid (Timmons and Fire, 1998) using the following primers: 5′-CGCGAAGCTTGCCGATTTGCCAGAAAAATGGA-3′ and 5′-CGCGGTCGACGACTGAGTTACTGTCACACGTGG-3′. For other genes, the *C. elegans* RNAi library (Source BioScience, Nottingham, UK) was used (Fraser et al., 2000). For *hmp-2* (RNAi) to measure the stable repulsion ratio (Fig. S5), a combination of feeding RNAi and injection RNAi was conducted in the LP316 strain. Injection RNAi was performed as described previously (Hara and Kimura, 2009), and double-stranded (ds)RNA was prepared using cenix:72-h4 as the template (the Phenobank database: http://worm.mpi-cbg.de/phenobank/cgi-bin/ProjectInfoPage.py). After injection, recovered worms were incubated on plates for feeding RNAi of *hmp-2* at 22°C for 36 to 40 h until use.

### Deformation of the *C. elegans* eggshell in microchambers

Procedures to deform embryos of the sea urchin (Chang et al., 2014; Minc et al., 2011; Tanimoto et al., 2016) were adopted to deform the *C. elegans* eggshell. A SU-8-positive master containing tens of posts that were 24 μm in height and of different shapes was first constructed by microlithography. A 10:1 mixture of PDMS Sylgard 184 silicone elastomer and curing agent (Dow Corning) was poured onto the master, and air bubbles inside the mixture were removed using a desiccator vacuum for 30 min; then, the mixture was baked at 65°C for 2 h. The replica was cut, peeled off the master, and activated with plasma cleaner (PDC-32G; Harrick Plasma, Ithaca, NY). *C. elegans* embryos were dissected from adult worms and collected in M9 buffer (Brenner, 1974). The two-cell stage embryos were transferred on to the micro chamber by using a mouth pipette and placed into the target chamber via manual handling using an eyelash bar in M9 buffer. A glass coverslip (18×18 mm^2^) was then placed on top of the solution, and buffer was gently sucked from the slides of the coverslip with a paper towel to slowly push the embryos into the chamber. The embryos were imaged at the four-cell stage.

### Eggshell removal

Eggshells were removed using a previously described method with modifications (Park and Priess, 2003). Embryos were treated with Kao bleach (Kao, Tokyo, Japan) mixed with 10 N KOH at a 3:1 ratio for 90 s, and placed in Shelton's growth medium (SGM) (Shelton and Bowerman, 1996) for washing three times. The vitelline membrane was removed by using a 30-μm micropipette made by pulling a glass capillary (GD-1; Narishige, Tokyo, Japan) with a micropipette puller (P-1000IVF; Sutter Instrument, Novato, CA). In order to investigate the role of Ca^2+^ (Fig. S4), after eggshell removal, the embryos were placed in Ca^2+^-free 0.75× egg salt (125 mM NaCl, 40 mM KCl, 3.4 mM MgCl_2_, 5 mM HEPES pH 7.2, 2 mM EDTA) or in 0.75× egg salt (118 mM NaCl, 40 mM KCl, 3.4 mM CaCl_2_, 3.4 mM MgCl2, 5 mM HEPES pH 7.2).

### Image acquisition

Embryos were placed in 0.75× egg salt (or in SGM if the eggshell was removed). For fluorescence imaging, embryos were visualized at room temperature (22–24°C) with a spinning-disk confocal system (CSU-X1; Yokogawa, Tokyo, Japan) mounted on an inverted microscope (IX71; Olympus, Tokyo, Japan) equipped with a 60×1.30 NA objective (UPLSAPO 60XS2; Olympus). Images were acquired with a CCD camera (iXon; Andor Technology, Belfast, UK) controlled by Metamorph software (version 7.7.10.0). Images are shown as maximum-intensity projections of planes spaced 1.0 μm apart. Images were analyzed with ImageJ (National Institute of Health, Bethesda, MD). To evaluate whether the eggshell was axially symmetric along the AP axis (Fig. S1D), embryos were placed in 20 μg/ml Texas Red-conjugated Dextran (Molecular Probes, D1864) in M9 buffer. Then, the ratio between the longer axis and the shorter axis of the cross-section perpendicular to the anterior-posterior axis was visualized using the Volume Viewer macro (developed by Dr Kai Uwe Barthel) for ImageJ, fitted to an ellipsoid by hand, and the AR was quantified with ImageJ.

To calculate *α*, phase contrast images acquired at room temperature under an inverted microscope (Axiovert 100; Carl Zeiss, Oberkochen, Germany) equipped with a 40×0.70 NA objective (Plan-Neofluar; Carl Zeiss) were used. Images were acquired with a CCD camera (ORCA-100; Hamamatsu, Hamamatsu, Japan) controlled by iVision-Mac (version 4.0.9; BioVision Technologies, Exton, PA). The AR and *α* were quantified using ImageJ. For quantification of *α*, each blastomere was fitted into a precise circle by hand, and *R_i_* and *d_ij_* were quantified. The parameter *d_ij_* was calculated from the centroids of the cells.

Embryos in the microchambers were imaged at room temperature using a microscope (BX51; Olympus) equipped with a 32×0.40 NA objective (PH1-Achrostigmat; Carl Zeiss). Images were acquired with a CCD camera (Orca-DCAM; Hamamatsu) controlled by IPLab (version 4.0.8; BD Biosciences, San Jose, CA). The AR was quantified with ImageJ.

### The orientations of cell division in *par-2* (RNAi) and *par-3* (RNAi) embryos

The orientation of cell division in AB and P1 cells was defined as the direction connecting the centers of a pair of daughter chromosomes immediately following the onset of anaphase. The chromosomes were visualized using strains expressing GFP::H2B –EG4601, CAL1661 (*dpy-11*), and CAL1671 (*lon-1*) strains (Table S2). The centers of chromosomes were quantified by using Imaris software (Bitplane, Zurich, Switzerland). The orientations of cell division were classified into four classes (Fig. 8A). In the T-div class, one cell divides perpendicularly (>45°) to the longest (AP) axis, while the other cell divides parallely (≤45°) to the AP axis. In the I-div class, both cells divide parallely (≤45°) to the AP axis. In the H-div and C-div classes, both cells divide perpendicularly (>45°) to the AP axis. In the H-div class, the orientations of the cell divisions of the two cells are parallel (≤45°), whereas in the C-div class, the orientations are perpendicular (>45°) to each other and are not in the same plane (≤45°). We found some cases (*n*=7/66) in which the orientations of cell divisions did not fall into the four classes. We did not include these cases in the analyses.

### Statistical analysis

To confirm normality, the Shapiro–Wilk test was used. To confirm homoscedasticity, an F-test was used. If both normality and homoscedasticity were confirmed, Student's *t*-test was used to compare means; if only normality was confirmed, Welch's *t*-test was used. In other cases, Wilcoxon's rank sum test was used to compare mean values. *P*<0.05 was considered to represent statistical significance. For these analyses, R (www.r-project.org) was used. The experiments were not randomized, and the investigators were not blinded to allocation during experiments and outcome assessment.

### Construction of the AA model based on the RO model and computer simulations

Three-dimensional simulations of cell motion within the confined space of the eggshell were constructed by modifying a simulation developed by Weiss and colleagues (Fickentscher et al., 2013). The mathematical models consider cells to be soft spherical balls, and the eggshell as a rigid ellipsoid. Cell configurations were calculated from an initial configuration at successive time steps. Attraction forces were added in this study. We changed the AR value from 1.0 to 4.0 in increments of 0.1, while maintaining a constant total eggshell volume. The parameters used are listed in Table S3. The simulations were programmed in MATLAB; the source code is available upon request.

The eggshell was considered to be an ellipsoid whose center was the origin and long axis was on the *x*-axis in coordinate; the length of the long axis was defined as *lx,* and the length of the short axis as *ly* (=*lz*)*.* The AR was calculated as *lx*/*ly*. Cells were considered to be spheres of radius *R_i_* (*i*=1, 2,…, *N*; with *N* denoting the total number of cells). The center of mass is represented by position **r**_i_.

Cells were assumed to move in a highly viscous environment, and the positions were calculated by an overdamped Langevin's equation, as follows:
(1)
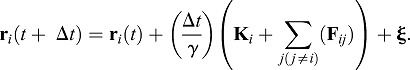
Random cell motion due to stochastic effects is represented by a vector ***ξ***. The components are three combinations of uncorrelated random numbers with a mean of 0 and variance of 0.027. The integration time step *Δt*=5 s.

#### Repulsive forces from the eggshell

When touching the eggshell, the cells experience a repulsive force defined as:
(2)
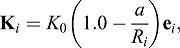
where **e***_i_* denotes a unit vector perpendicular to the eggshell, pointing into the cells, and *a* is the minimum distance between the center of cell *i* and the eggshell. *a* was calculated as the minimum solution of the following equation that determines the distances from a coordinate within the ellipsoid to the nearest edge of the ellipsoid. The equation is derived when we query the components of the vector perpendicular to the tangent plane on the ellipsoid.
(3)
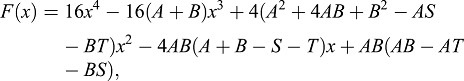
where *A* denotes the square of *lx*, *B* denotes the square of *ly*, *S* denotes the square of the *x* coordinate of the cell, and *T* denotes the sum of squares of the *y* and *z* coordinates of the cell. *a* was calculated using a function available in MATLAB; for *a*>*R*_i_, **K_i_=0**.

#### Repulsive and attractive forces between cells

The force between a pair of cells depends on the distance between the centers of the cells (cell *i* and cell *j*), *d_ij_*=|**r***_i_*−**r***_j_*|. For *d_ij_*≤*α*(*R_i_*+*R_j_*) two cells repel each other; for *α*(*R_i_*+*R_j_*)<*d_ij_*≤(*R_i_*+*R_j_*), two cells attract each other. Otherwise, the pairwise force is zero. Specifically, forces between any two cells were calculated as follows.

For 0<*d_ij_*≦min(*R_i_*, *R_j_*), **F*_ij_***=*F*_0_**e*_ij_*** and for min(*R_i_*, *R_j_*)<*d_ij_*≦0.5(1+*α*)(*R_i_*+*R_j_*),
(4)
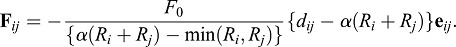
For 0.5(1+*α*)(*R_i_*+*R_j_*)<*d_ij_*≦(*R_i_*+*R_j_*),
(5)
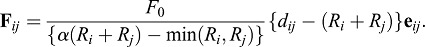
Otherwise, **F***_ij_*=**0**.

Here, **e***_ij_*=**d***_ij_*/|**d***_ij_*| is the unit vector pointing from cell *i* to cell *j*. *R_i_* and *R_j_* represent the radii of cell *i* and cell *j*, respectively. *α* is the parameter at which the pairwise force is zero between two attached cells. In the expressions, min() represents the minimum value of the parameter in parentheses. The constant force for *0<d_ij_<*min(*R_i_, R_j_*) reflects cell elongation during cytokinesis, which occurs roughly at a constant velocity.

#### Cell divisions

The orientations of the division axes of the first (P0 to AB and P1), second (AB to ABa and ABp), and third division (P1 to EMS and P2) are parallel to the *x*-, *z*- and *x*-axis, respectively. When we modeled the cell arrangement for *par-2* (RNAi) and *par-3* (RNAi) embryos (Fig. 8), the following orientations were examined: for T-div, one cell divided along the *x*-axis, while the other divided along *z*-axis. For I-div, both cells divided along the *x*-axis. For H-div, both cells divided along the *z*-axis. For C-div, one cell divided along the *z*-axis, while the other divided along the *y*-axis.

#### Initial configuration

At the initial time step, two cells are positioned at the center of the eggshell 2.0-μm apart on the *x*-axis, as the orientation of the first cell division is parallel to the long axis of the embryo.

#### Classification of the cell arrangement patterns

The patterns of cell arrangements were classified based on whether the cell–cell distance (*d_ij_*) was smaller or larger than the sum of radii (*R_i_*+*R_j_*) in each combination of two cells. When the cell–cell distance was smaller than the sum of radii, the cells were considered to be in contact each other. The simulation was repeated for 30 times for each condition.

## Supplementary Material

Supplementary information

Supplementary information
