# Supplementary material for: An asymmetric attraction model for the diversity and robustness of cell arrangement in nematodes
Source: Development. 2017 Dec 1;144(23):4437–49. doi: 10.1242/dev.154609 (PMC5769634; doi:10.1242/dev.154609)
Supplement: Supplementary information [file develop-144-154609-s1.pdf]

Supplementary Tables

Table S1. AR and pattern of cell arrangement in different nematode species

genera/species	aspect ratio	pattern	references
Enoplus brevis	1.17	Pyramid	(Schulze and Schierenberg, 2011)
Halicephalobus	1.37	Diamond	(Goldstein, 2001)
PS1010	1.39	Diamond	(Goldstein, 2001)
Goodeyus	1.39	Diamond	(Goldstein, 2001)
Caenorhabditis	1.40	Diamond	(Goldstein, 2001)
Bunonema	1.45	Diamond	(Goldstein, 2001)
Rhabditis	1.45	Diamond	(Goldstein, 2001)
Teratorhabditis	1.51	Diamond	(Goldstein, 2001)
Plectonchus	1.62	Diamond	(Goldstein, 2001)
Cruznema	1.62	Diamond	(Goldstein, 2001)
Panagrellus	1.67	Diamond	(Goldstein, 2001)
Mesorhabditis	1.76	Diamond	(Goldstein, 2001)
Panagrobelus	1.77	Diamond	(Goldstein, 2001)
Pristionchus	1.77	Diamond	(Goldstein, 2001)
Aduncospiculum	1.78	Diamond	(Goldstein, 2001)
Nothacrobeles	1.84	Diamond	(Goldstein, 2001)
Diploscapter	1.88	Linear	(Goldstein, 2001)
Diploscapter coronata	1.90	Linear	This study
Acrobeloides	1.91	T-shaped	(Goldstein, 2001)
Protorhabditis	1.91	Linear	(Goldstein, 2001)
Cephalobus	1.97	T-shaped	(Goldstein, 2001)
Rhabditella	2.02	Diamond	(Goldstein, 2001)
Eucephalobus	2.03	T-shaped	(Goldstein, 2001)
Pseudoacrobeles	2.06	T-shaped	(Goldstein, 2001)
Panagrolaimus	2.09	Diamond	(Goldstein, 2001)
Cervidellus	2.18	T-shaped	(Goldstein, 2001)
Acrobeles	2.21	T-shaped	(Goldstein, 2001)
Diplenteron	2.31	Diamond	(Goldstein, 2001)

Development • Supplementary information

Aphelenchoides	2.44	T-shaped	(Goldstein, 2001)
Meloidogyne	2.47	Linear	(Goldstein, 2001)
Zeldia	2.47	Linear	(Goldstein, 2001)
Chiloplacus	2.48	T-shaped	(Goldstein, 2001)
Nacobbus	2.52	Linear	(Goldstein, 2001)
Pratylenchus	2.56	Linear	(Goldstein, 2001)
Teratocephalus	2.69	Diamond	(Goldstein, 2001)
Aphelenchus	2.79	Diamond	(Goldstein, 2001)
Belonolaimus	4.11	Linear	(Goldstein, 2001)
Aphelenchoides besseyi	4.40	Linear	This study

References

Goldstein, B. (2001). On the evolution of early development in the Nematoda. *Philos. Trans. R. Soc. Lond. B Biol. Sci.* **356**, 1521–1531.

Schulze, J. and Schierenberg, E. (2011). Evolution of embryonic development in nematodes. *Evodevo* **2**, 18.

Table S2. ***Caenorhabditis elegans*** **strains used in this study**

name	genotype	sources
N2	wild type	CGC*
CB185	*lon-1*(*e185*)III	CGC
CB207	*dpy-11*(*e207*)V	CGC
EG4601	*oxIs279* [*pie-1p::GFP::H2B* + *unc-119*(+)]II; *unc-119*(*ed3*)III	CGC
FT741	*xnSi6* [*mex-5P::hmr-1::GFP::hmr-1 3′-UTR* + *unc-119*(+)]II; *unc-119*(*ed3*)III	CGC
GR1034	*ceh-18*(*mg57*)X	CGC
LP172	*hmr-1*(*cp21*[*hmr-1::GFP* + *LoxP*]) I.	CGC
LP316	*hmp-2*(*cp78*[*GFP::hmp-2a* + *LoxP*]) *III.*	CGC
RB1353	*spv-1*(*ok1498*)II	CGC
CAL1661	*dpy-11*(*e207*)V; *oxIs279* [*pie-1p::GFP::H2B* + *unc-119*(+)]II; *unc-119*(*ed3*)III	This study
CAL1671	*lon-1*(*e185*)III; *oxIs279* [*pie-1p::GFP::H2B* + *unc-119*(+)]II; *unc-119*(*ed3*)III	This study

* CGC = *Caenorhabditis* Genetics Center

Table S3. Parameters implemented in the computer simulation

parameters		values	references
Force			
Force by the eggshell	K_0/γ	0.2 (μm/sec)	(Fickentscher et al., 2013)
Force by cells	F_0/γ	0.1 (μm/sec)	(Fickentscher et al., 2013)
Random fluctuation	ξ	0 ± 0.027(μm)	(Fickentscher et al., 2013)
Stable repulsion ratio	α	0.7–1.0	This study
Time			
Time interval per step	Δt	5 (sec)	(Fickentscher et al., 2013)
Duration time			
at 2-cell stage		186 (step)	(Fickentscher et al., 2013)
at 3-cell stage		72 (step)	(Fickentscher et al., 2013)
at 4-cell stage		172 (step)	(Fickentscher et al., 2013)
at 3-cell stage (*par-2*, *par-3* RNAi*)		0 (step)	This study
at 4-cell (*par-2*, *par-3* RNAi)		156 (step)	This study
Size and Geometry			
Aspect ratio of the eggshell	*AR*	1.0–4.0	This study
Total volume of cells	*Volume*	23,550 (μm^3)	(Fickentscher et al., 2013)
Cell radius			
of AB cell	*R_AB*	15.00 (μm)	(Fickentscher et al., 2013)
of P1 cell	*R_P1*	13.10 (μm)	(Fickentscher et al., 2013)
of ABa cell	*R_ABa*	11.90 (μm)	(Fickentscher et al., 2013)
of ABp cell	*R_ABp*	11.90 (μm)	(Fickentscher et al., 2013)
of EMS cell	*R_EMS*	11.05 (μm)	(Fickentscher et al., 2013)
of P2 cell	*R_P2*	9.65 (μm)	(Fickentscher et al., 2013)
at 2-cell stage (*par-2*, *par-3* RNAi**)	*R_2cell*	14.12 (μm)	This study
at 4-cell (*par-2*, *par-3* RNAi**)	*R_4cell*	11.20 (μm)	This study

* Under the *par-2* and *par-3* (RNAi) conditions, the two cells at the 2-cell stage divide simultaneously, and there is no three-cell stage.

** Under the *par-2* and *par-3* (RNAi) conditions, there are no asymmetry in volume at the 2-cell or 4-cell stage.

Reference

Fickentscher, R., Struntz, P. and Weiss, M. (2013). Mechanical Cues in the Early Embryogenesis of Caenorhabditis elegans. *Biophys. J.* **105**, 1805–1811.

Supplemental Figures

Figure S1

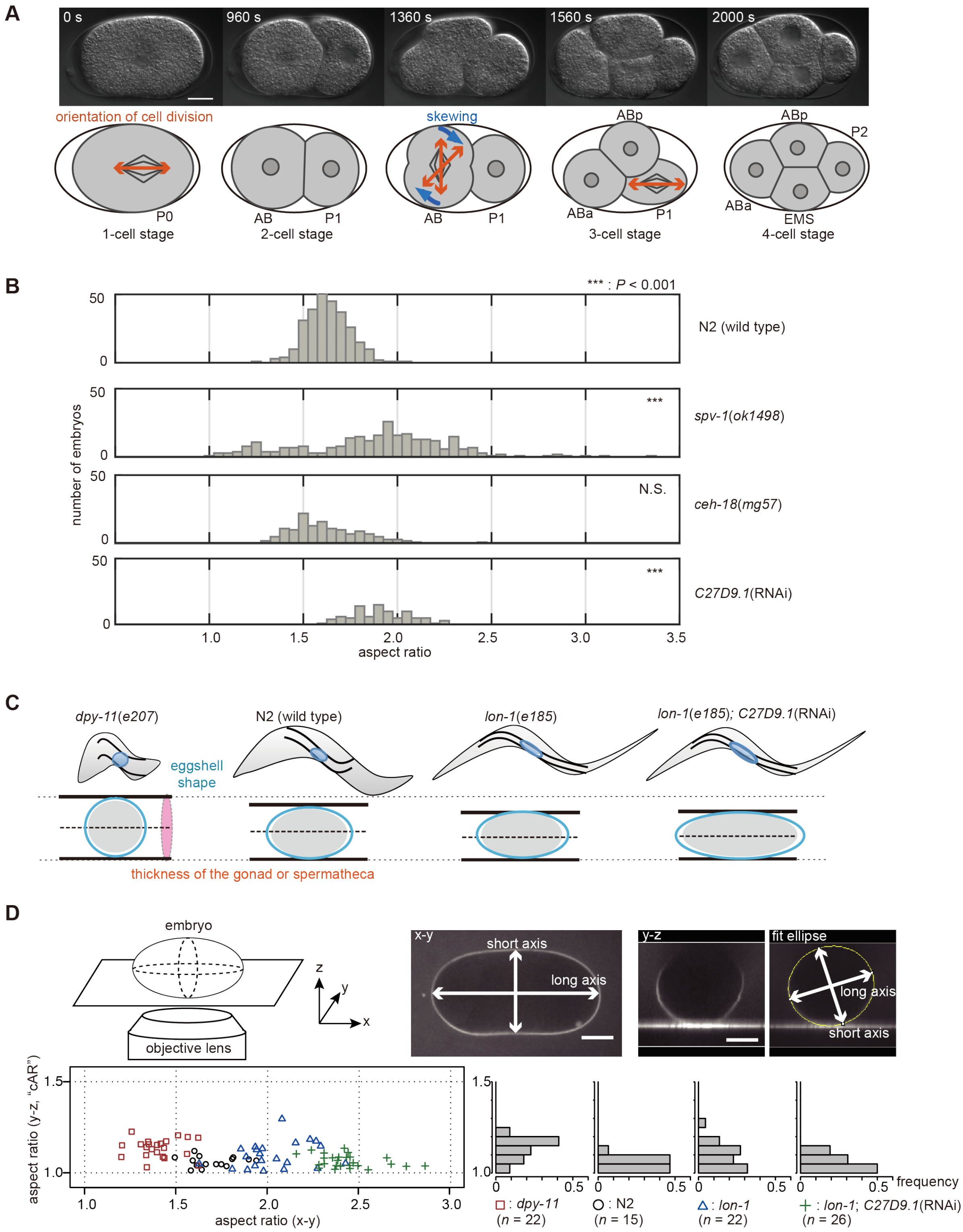

Development • Supplementary information

Figure S1. Eggshell shapes in mutant and RNAi-treated strains of *C. elegans* and the model that explains the variation in eggshell shapes

(A) The diamond-type cell arrangement is formed in *C. elegans* embryo as follows: At the one-cell stage, the P0 cell divides parallel to the anterior-posterior axis. The anterior daughter cell of P0 is named AB, and the posterior daughter cell is P1. AB divides vertically to the anterior-posterior axis, and during cell elongation, the orientation of cell division axis is skewed. The anterior daughter cell of AB is named ABa, and the posterior daughter cell ABp. Then, P1 divides parallel with the anterior-posterior axis. The daughter cell of P1 in contact with ABa and ABp is named EMS. The posterior daughter cell of P1 is named P2. At the 4-cell stage, all cells, except ABa and P2, are in contact with each other. Theoretically, the diamond-type-arrangement with ABa-P2 contact (and loss of ABp-EMS contact) is possible. However, such an arrangement has never been observed experimentally throughout this study. (B) Histograms showing ARs in *C. elegans* mutants and RNAi-treated strains. In a previous study, an *spv-1* mutant was reported to produce embryos of various shapes (Tan and Zaidel-Bar, 2015). We did not use the *spv-1* mutant for the other part of the present study as this strain produces embryos with non-ellipsoidal shape and/or with very small or very large volumes. The means ± SDs of the eggshell shapes in each strain are as follows: N2 (1.6 ± 0.1, $n = 281$), *spv-1*(*ok1498*) (1.9 ± 0.4, $n = 283$), *ceh-18*(*mg57*) (1.6 ± 0.2, $n = 183$), *C27D9.1* (RNAi) (1.9 ± 0.2, $n = 90$). For statistical analysis, normality was tested with a Shapiro–Wilk test, and homoscedasticity was confirmed using an F test; ***$P < 0.001$ vs. N2 (wild type). Student's *t*-test was used for*C27D9.1* (RNAi). Wilcoxon's rank-sum test was used for *spv-1*(*ok1498*), and *ceh-18*(*mg57*). (C) A model that explains how eggshell shape is defined: The following two factors are considering determine eggshell shape: (1) the cross-sectional area of the gonad and (2) oocyte volume; *dpy-11* mutants are considered to have thick gonads and *lon-1* mutants slender gonads. Increased volume of oocyte induced by *C27D9.1* (RNAi) might be squeezed in the gonad. (D) Quantification of the aspect ratio of the cross-sectional area (cAR) of the eggshell perpendicular to the long axis; upper left panel shows the definition of axes. The x-y plane is parallel to the focal planes of the microscope. Upper middle and right panels are images of the eggshell visualized by Texas Red-Dextran staining (bar = 10 μm). The white lines with arrows represent long and short axis of the eggshells in x-y (upper middle) or y-z (upper right) plane. Lower left panel is the plot of cAR against aspect ratio of x-y plane, and lower right panels are histograms of cAR. The measurements were performed in four kinds of *C. elegans* strains. N2 (black circle, n = 15), *dpy-11*(*e207*) (red square, $n = 22$), *lon-1*(*e185*) (blue triangle, $n = 22$), *lon-1*(*e185*);

Development • Supplementary information

C27D9.1(RNAi) (green cross, *n* = 26).

Reference

Tan, P. Y. and Zaidel-Bar, R. (2015). Transient membrane localization of SPV-1 drives cyclical actomyosin contractions in the C. elegans spermatheca. *Curr. Biol.* **25**, 141–151.

Development • Supplementary information

Figure S2

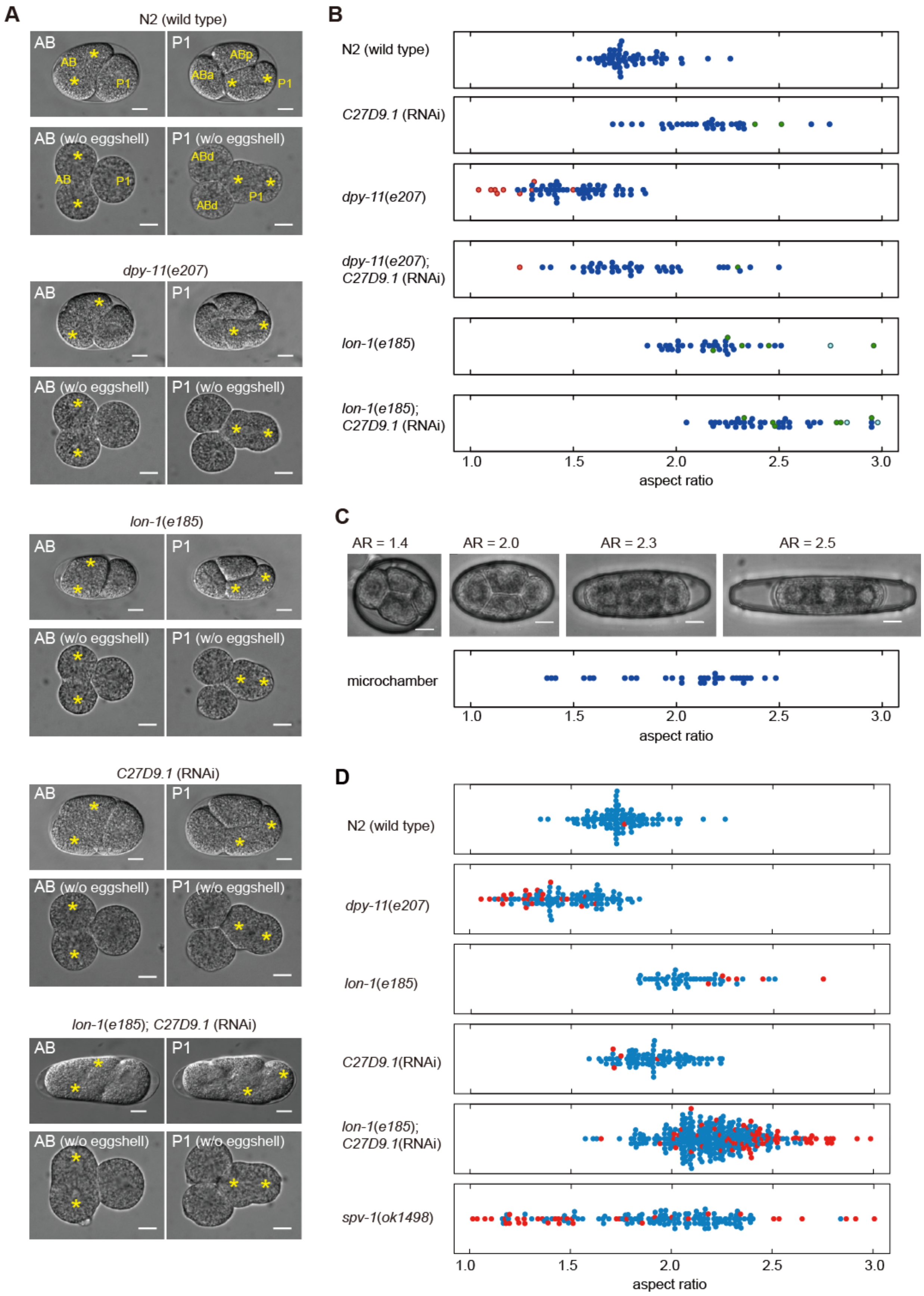

Development • Supplementary information

Figure S2. Blastomere configuration in embryos of *C. elegans* after removal of the eggshell and the relationship of cell arrangement patterns and hatching rate with the ARs in various strains of *C. elegans*

(A) The orientation of cell division in N2 (wild type), *dpy-11*(*e207*), *lon-1*(*e185*), *C27D9.1* RNAi-treated strains with N2 or *lon-1*(*e185*) background (upper panels): The orientations are easier to observe in embryos with the eggshell removed at the 1–2-cell stage and cultured until the 4-cell stage (lower panels). The daughter cells of the AB cell (ABa and ABp, or ABd in eggshell removed embryos) divide vertically (left), and the daughter cells of the P1 cell (EMS and P2) divide horizontally (right). Spindle poles are represented by yellow asterisks. Scale bars are 10 µm. (B) Bee swarm plots displaying the relationship between each of the four types of cell arrangement (blue, diamond type; red, pyramid type; green, T-shaped type; cyan, linear type) and the ARs of eggshells in N2 (wild type) (*n* = 52), *dpy-11*(*e207*) (*n* = 59), *lon-1*(*e185*) (*n* = 36), *lon-1*(*e185*); *C27D9.1* (RNAi) (*n* = 41) (C) Upper micrographs show images of N2 embryos in microchambers (scale bars = 10 µm). Lower panel indicates the bee swarm plot showing the distribution of ARs in the microchambers (n = 32). All embryos adopted the diamond arrangement (blue dots) under these conditions. It should be noted that, with this mechanical confinement experiment, we were not able to change the AR below 1.2 or above 2.8, where the cells are expected to take the other arrangements than the diamond type. (D) ARs and embryonic development (hatched (blue) or not hatched (red)) were examined in N2 (wild type) (*n* = 98), *dpy-11*(*e207*) (*n* = 109), *lon-1*(*e185*) (*n* = 53), *C27D9.1* RNAi-treated strain with the N2 (*n* = 90) or *lon-1*(*e185*) (*n* = 322) background, and *spv-1*(*ok1498*) (*n* = 150).

Development • Supplementary information

Figure S3

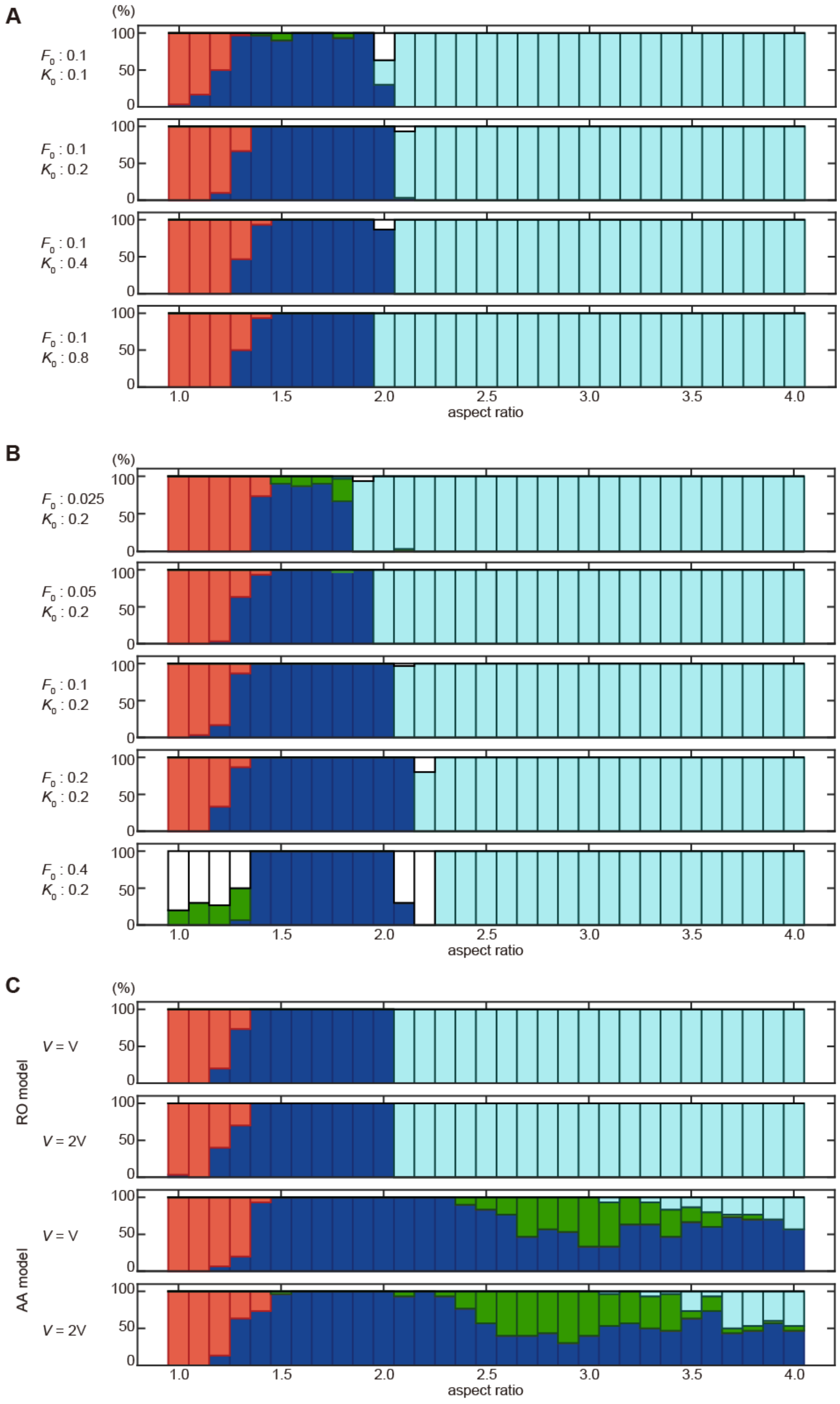

Development • Supplementary information

Figure S3. Simulation of cell arrangement patterns for various sets of parameters

(A, B) Relationship between rates of appearance of various cell arrangement patterns (blue, diamond type; red, pyramid type; green, T-shaped type; cyan, linear type; white, others) and the ARs in the RO model under changing force strength (cell–eggshell; K_0 (A), cell–cell; F_0 (B)). We focused on the parameter of repulsive force strength, as the other parameters in this model were based on experimental measurements. Moreover, because the absolute values of the forces affect only division speed but not the final (stable) position of the cell, we changed the ratio of repulsion forces between cell–cell (F_0) and cell–eggshell (K_0). We first determined the range for cells acquiring the diamond-type arrangement in a normal shape. Next, within this ratio range, we changed the shape and examined whether diversity and robustness were reproduced. The trend in frequency patterns of cell arrangements did not change greatly. (C) The cell volume parameter was changed two-fold to mimic *C27D9.1* (RNAi) conditions in embryos in the RO model and the AA model; the parameter did not affect the overall tendency of the distribution. From the measurement of the long and short axes of the embryo *in vivo*, we estimated the volume of embryo to be ~2-fold larger in *C27D9.1* (RNAi).

Figure S4

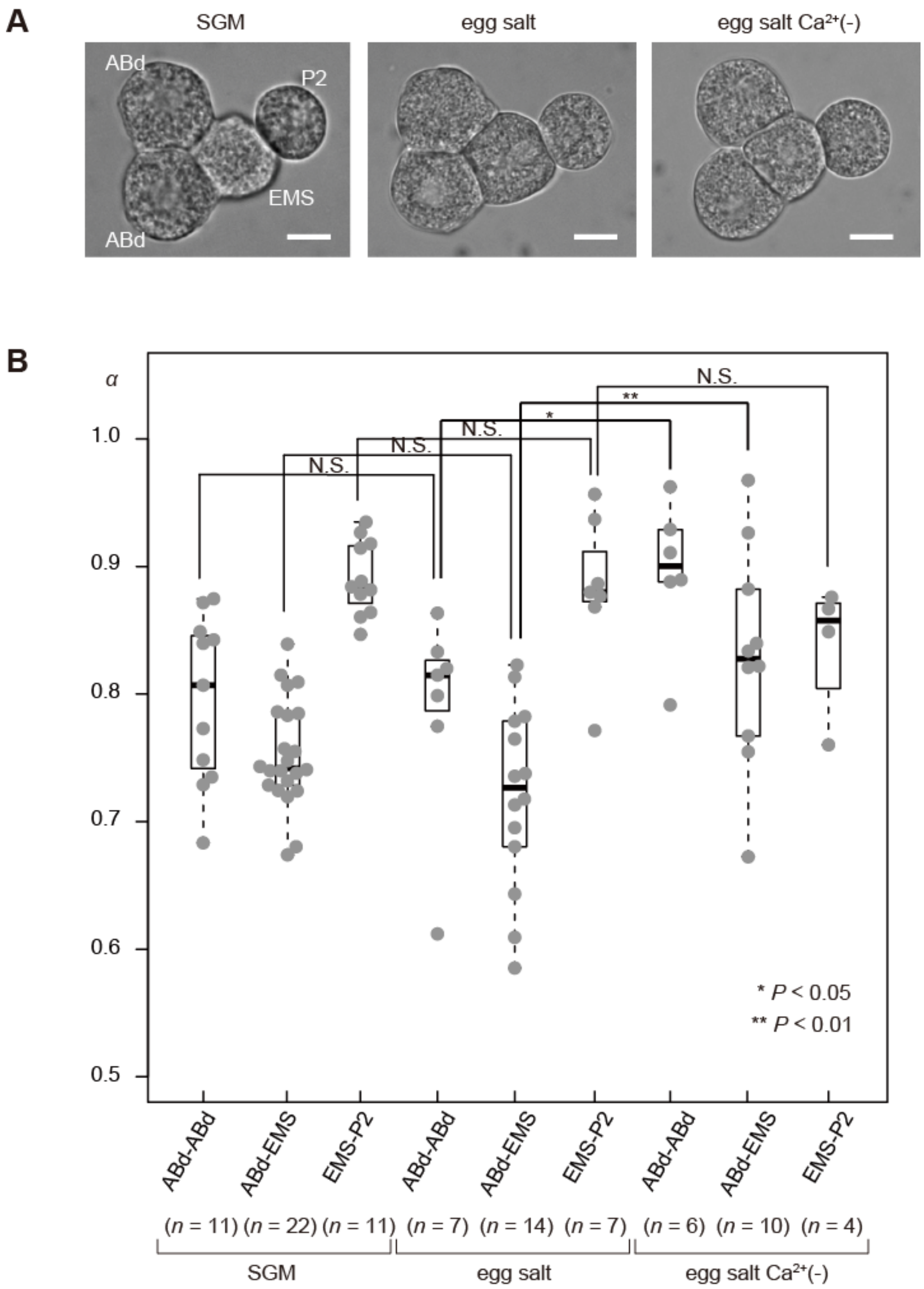

Figure S4. The attraction between the blastomeres was reduced in Ca^{2+}-free buffer

(A) Micrographs showing *C. elegans* embryos with eggshells removed, at the 4-cell stage in SGM (Shelton's growth medium), 0.75×egg-salt buffer, and Ca^{2+}-free-0.75×eggsalt buffer. Scale bars are 10 µm. The daughter cells of the AB cell were indicated 'ABd' as we were unable to distinguish between ABa and ABp cells when the eggshell was removed. (B) Bee swarm plot and boxplot of *α* in each combination of cell types AB daughter cells, AB daughter and EMS cells, and EMS and P2 cells in SGM, 0.75×egg-salt buffer, and Ca^{2+}-free-0.75×egg-salt buffer, respectively; *$P < 0.05$ and

Development • Supplementary information

**$P < 0.01$, Student's *t*-test for ABd-EMS in SGM and ABd-EMS in egg-salt pair, ABd-EMS in egg-salt and ABd-EMS in Ca^{2+}-free-egg-salt pair, EMS-P2 in egg-salt and EMS-P2 in Ca^{2+}-free-egg-salt pair. Welch's *t*-test for EMS-P2 in SGM and EMS-P2 in egg-salt pair. Wilcoxon rank-sum test for ABd-ABd in SGM and ABd-ABd in egg-salt pair, ABd-ABd in egg-salt, and ABd-ABd in Ca^{2+}-free-egg-salt pair.

Development • Supplementary information

Figure S5

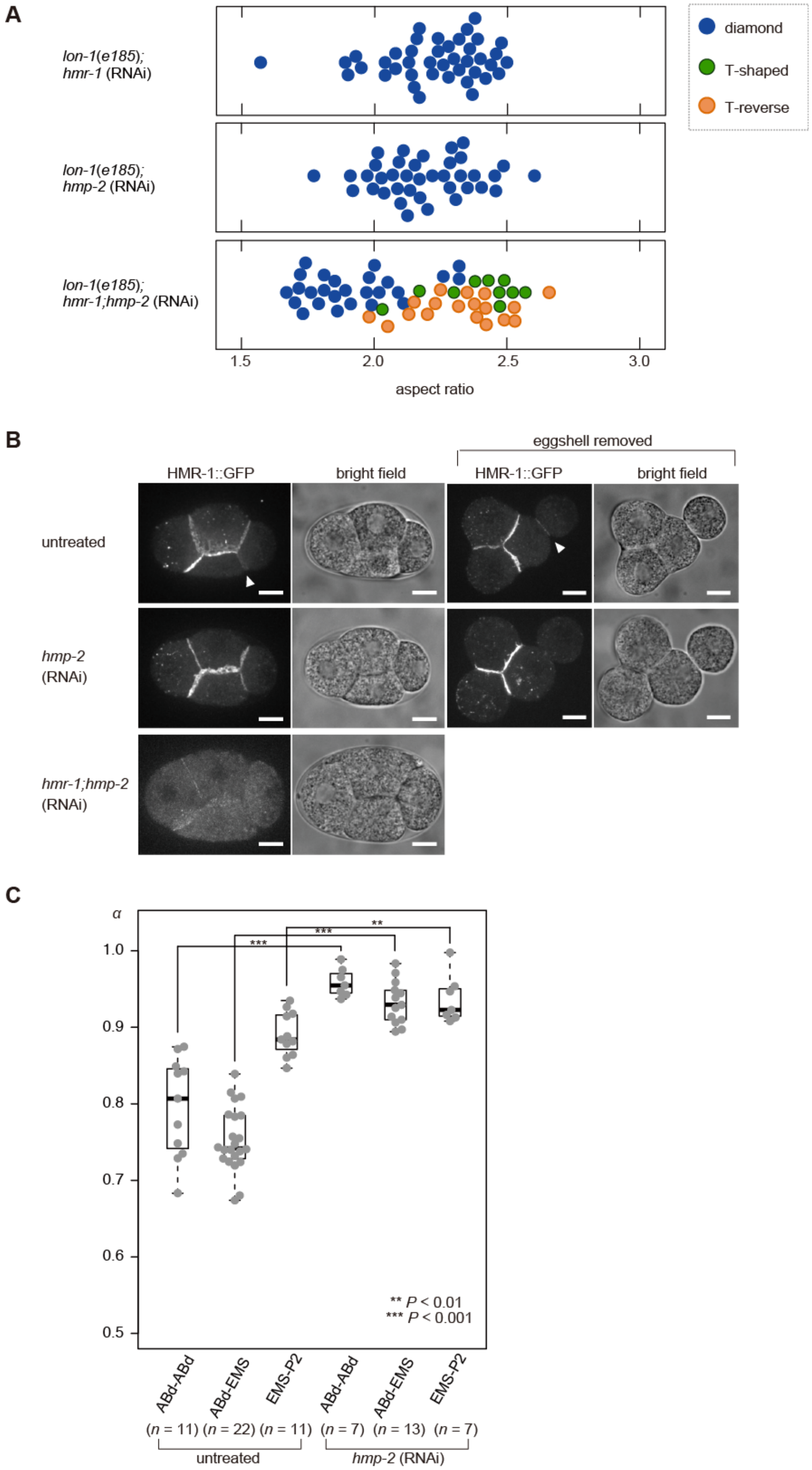

Development • Supplementary information

Figure S5. Knockdown of cell adhesion molecules impairs the robustness of the diamond-type cell arrangement against deformation in the *C. elegans* embryo

(A) Bee swarm plots displaying the relationship between different types of cell arrangement and the ARs for *hmr-1* (n = 41), *hmp-2* (n = 38), and *hmr-1*; *hmp-2* (n = 54)-knockdown strains with *lon-1*(*e185*) mutant background. For *hmr-1*- and *hmp-2*-knockdown strains, we selected and examined high-AR embryos to investigate their cell arrangements. For the *hmr-1*; *hmp-2*-knockdown strain, we examined lower-AR embryos as well. (B) Micrographs of embryos expressing HMR-1 fused with GFP protein (FT741 strain) in untreated, *hmp-2* RNAi-, and *hmr-1*; *hmp-2* RNAi-treated embryos with or without eggshell: We could not reproducibly obtain blastomeres without eggshell of *hmr-1*; *hmp-2*-RNAi-treated embryos because these embryos were damaged during the eggshell removal process. Scale bars are 10 µm. (C) Bee swarm plot and boxplot of α of untreated and *hmp-2*-RNAi-treated embryos. For statistical analysis, normality was tested with the Shapiro–Wilk test, and homoscedasticity was confirmed using an F test. Asterisks represent statistical significance; **$P < 0.01$, ***$P < 0.001$, Welch's *t*-test for ABd-ABd pair, ABd-EMS pair, and EMS-P2 pair.

Development • Supplementary information
